# The Widening Cross-Country Food Availability Gap

**DOI:** 10.3390/foods15203678

**Published:** 2026-10-16

**Authors:** Enes Çatı, Orhan Gündüz, Ufuk Gültekin

**Affiliations:** 1 Department of Agricultural Economics, Faculty of Agriculture, Malatya Turgut Özal University, 44210 Malatya, Türkiye; orhan.gunduz@ozal.edu.tr; 2 Department of Agricultural Economics, Faculty of Agriculture, Cukurova University, 01250 Adana, Türkiye; ugultek@cu.edu.tr

**Keywords:** dietary energy supply adequacy, food availability, food security monitoring, cross-country divergence, political stability, import dependency, SDG 2

## Abstract

National food supplies have improved almost everywhere since 2000, yet progress towards Zero Hunger has stalled. This study examines how the cross-country distribution of food availability evolved and what contributed to its renewed widening, using the FAO indicator of average dietary energy supply adequacy. For 150 countries observed from 2001 to 2023, dispersion declined rapidly through the late 2000s, remained relatively stable until the mid-2010s, and subsequently increased. A continuous three-segment model outperforms both a two-segment V and a constrained plateau model (ΔAICc = 39.75 and 12.10, respectively) and is selected in 98.7% of three-year moving-block bootstrap samples. The three-phase pattern is also recovered from the latest FAOSTAT vintage, although historical revisions affect individual estimates. Most renewed widening occurs within geographic regions and subregions, while 93% of the 2013–2023 increase in the 90th–10th percentile gap originates below the median. Countries initially occupying the lowest adequacy quintile improved faster than those in the highest, but transition matrices provide no evidence of increasing mobility. Political stability and adequacy exhibit contemporaneous co-movement, although this association cannot be interpreted causally. Food-security monitoring should complement national and regional averages with distributional indicators that capture cross-country disparities. The concentration of renewed divergence within regions and in the lower half of the distribution suggests that food-security policies should target country-specific vulnerabilities rather than rely solely on broad regional classifications. In particular, tracking countries that persistently remain in or newly enter the lowest adequacy group could help prioritise support and better target food-security interventions. Overall, the findings highlight the importance of distribution-sensitive monitoring and policy approaches for advancing SDG 2 without assuming a universal turning year or a common trajectory across countries.

## 1. Introduction

Two accounts of global food security sit uneasily together. The first is one of sustained progress: dietary energy supply per person has risen across most of the world since 2000, and national food supply now exceeds average requirements in the great majority of countries. The second is one of stalled progress: the number of people facing hunger has risen since the middle of the last decade, and Zero Hunger is described as off track [1]. These accounts are usually reconciled by pointing to particular disturbances—the food price episodes of 2007–2008 and 2010–2011, the COVID-19 disruption, and the war in Ukraine [2,3,4,5]—each of which raised poverty and worsened household food security where it landed [6,7,8,9,10].

A further reconciliation has not been examined. A world in which every country improves is not the same as a world in which the average improves because the already well-supplied improve faster. The statistics that dominate food security monitoring—levels, global totals and regional averages—do not distinguish the two. What distinguishes them is how national outcomes are spread out, and that spread is not routinely reported.

The question is worth asking because the policy target is distributional. An agenda expressed as leaving no one behind is not satisfied by a rising mean. The spread also carries information about timing: if countries stopped growing more alike when world food prices first spiked, that points to a different diagnosis than if they stopped when the pandemic began.

Where this question has been put to other development outcomes, it has changed the picture. Across Indian states, life expectancy at birth grew steadily more alike while infant mortality, neonatal mortality and fertility pulled apart after 2006 [11]. Across European regions, the worst-performing regions caught up with the leaders, yet the spread between regions did not narrow [12]. Food-system studies document increasing similarity in crop composition [13] and declining cross-country inequality in several dietary-energy and nutrient measures through 2010, alongside concentration of undernutrition in a smaller set of countries [14]. Prices, trade exposure and conflict can nevertheless transmit asymmetric shocks across countries [7,8,9,10,15,16,17,18,19,20,21,22,23,24,25,26,27,28,29], while resilience depends on coordinated agricultural, climatic, land-use and socioeconomic adaptation [30] and on water allocation and crop-specific adaptation under uncertainty [31,32,33]. This literature establishes convergence in composition and earlier food-system outcomes, but leaves open whether the level of energy-supply adequacy continued to converge after the global food-price crisis.

The indicator used here is average dietary energy supply adequacy, which expresses a country’s dietary energy supply as a percentage of its estimated average requirement. It belongs to the availability dimension of food security rather than to access or utilisation [34]; the distinction matters because the two need not move together. Across affluent countries, food availability and food access have been found to be unrelated [35]. Adequacy has served as an outcome variable in cross-country food security research, both on its own [36] and alongside average protein supply in panel analyses of trade and food security [37]; food balance sheet indicators of this kind have also been used to characterise national nutrition security and import reliance [38].

This study applies dispersion and convergence measures to the FAO country panel to document how the international distribution of food-availability levels has changed. The contribution is narrower than the broader literature on dietary and crop-composition homogenisation [13]: it evaluates convergence in the adequacy level, compares alternative temporal shapes, decomposes subsequent widening within and between geographic groups, and distinguishes movement of distributional positions from movement of named countries.

Two integrated questions are addressed. First, how did the cross-country distribution of food availability evolve between 2001 and 2023, when did its phases change, and how sensitive is that timing to scale, weighting, data vintage and influential countries? Second, how is renewed widening composed across regions, distributional tails and named countries, and which observed country characteristics co-move with it? These questions organise the analysis around temporal evolution and the composition of divergence rather than five separate tests.

## 2. Materials and Methods

### 2.1. Indicators and Data

Six indicators were drawn from the FAOSTAT Suite of Food Security Indicators. The primary indicator is average dietary energy supply adequacy, expressed as a percentage of the average dietary energy requirement. Adequacy was preferred because it is continuous over a wide range and is published for a large annual panel. The comparison indicators are the prevalence of undernourishment and the prevalence of moderate or severe food insecurity. Three additional supply indicators are average protein supply, animal-protein supply and per capita food-supply variability. The analytical snapshot was archived with the replication package. FAO introduced a revised Food Balance Sheet methodology for 2010 onward and has subsequently recompiled historical series using revised conversion factors and population estimates [39]. We therefore treat 2010 as a potential measurement boundary and report a complete re-extraction from the latest available FAOSTAT release as a data-vintage sensitivity test.

FAOSTAT publishes adequacy and undernourishment as overlapping three-year averages; each observation was assigned to the midpoint of its labelled interval. Consecutive observations therefore share two component years and are intrinsically serially dependent. The latest window used in the submitted snapshot is labelled 2022–2024 and is assigned to 2023; it is an estimate rather than a single-year observation. In the latest FAOSTAT release, only 33.5% of reporting areas have the same value in 2021–2023 and 2022–2024, so these windows are not treated as interchangeable. The prevalence of undernourishment is published as ‘<2.5%’ at the lower bound. For numerical calculations this category was coded at its midpoint, 1.25%; sensitivity analyses also use 0%, 2.5%, and exclusion of countries ever subject to suppression.

The adequacy indicator is published rounded to whole numbers. The 3450 country-year observations of the balanced panel contain 94 distinct values; 31.4% of annual changes are exactly zero and 72.9% are one percentage point or less. The measurement grid is therefore one percentage point. This matters for statistics computed on individual percentiles and is the reason why, in Section 3.4, the lower tail is characterised by country groups rather than by the movement of a single percentile.

The country characteristics used in Section 3.6 are the cereal import dependency ratio and the value of food imports in total merchandise exports from FAOSTAT, GDP per capita from the World Development Indicators, and political stability together with the mean of government effectiveness, control of corruption and rule of law from the Worldwide Governance Indicators [40]. Population-weighted statistics use fixed 2008 totals from the World Development Indicators, matched for all 150 countries. The year 2008 was originally chosen because it is the baseline for the fixed-country change and quintile analyses, not because it is assumed to be demographically typical. Because it coincides with the global food-price crisis, we repeated the weighted dispersion analysis using fixed population totals from 2001, 2013 and 2023. All four fixed-weight schemes attain their minimum in 2013 and show the same subsequent direction; the 2023-to-pre-minimum recovery share ranges from 1.39 to 1.42. A contemporaneous-weight estimate is also reported as a contrast, but it mixes distributional change with changing population shares. The fixed-weight result is therefore not specific to the 2008 population structure.

### 2.2. Samples

Convergence statistics respond mechanically to changes in sample composition, so balanced panels were used throughout. Requiring an unbroken annual series over 2001–2023 yields 150 countries and 3450 observations for adequacy and 159 countries and 3657 observations for undernourishment. The experience-scale series begins later; requiring an unbroken series from 2015 yields 92 countries over nine years. Table 1 summarises the samples. An unbalanced sample of countries observed in at least 18 years was used as a check.

### 2.3. Convergence Measures and Turning Point

Two questions were put to the distribution. Beta-convergence asks whether countries that started worse off improved faster; sigma-convergence asks whether the cross-country spread narrowed. A negative beta coefficient can coexist with rising sigma dispersion because beta summarises the conditional mean relationship between initial level and subsequent growth, whereas sigma also responds to heterogeneous shocks, changing ranks and residual variance [41,42]. Beta-convergence was estimated from average annual log growth and is reported as a descriptive complement, not as proof of falling dispersion.

$$\frac{-\beta }{100}=\frac{\left(1-e^{-\lambda T}\right)}{T},\;t_{1/2}=\frac{ln\;2}{\lambda }$$

where β is the estimated beta coefficient, T the length of the sub-period and t½ the half-life; the left-hand side is positive because β is negative. These are the durations implied by point estimates, and no confidence interval was computed for them.

Sigma-convergence was measured as the cross-country standard deviation of the indicator in each year. Adequacy is already a ratio of supply to requirement, so the raw scale is the natural one; because the cross-country mean rises by 9.6% over the period, a logarithmic transformation deflates dispersion as the mean grows, and both scales are reported unweighted and population-weighted. Three further measures guard against sensitivity to the tails: the interquartile range over the median, the difference between the 90th and 10th percentiles, and the coefficient of variation. The cross-country variance of log adequacy was also decomposed into between-region and within-region components using the seven-region United Nations M49 grouping: total variance is (1/N)Σ(v_i − $\bar{v}$)^2^, the between-region component is (1/N)Σ_g n_g($\bar{v}$_g − $\bar{v}$)^2^, and the within-region component is the difference between them, so the decomposition is exact. Countries are equally weighted and regions are weighted by the number of countries they contain.

The distribution was examined from two angles. The view that tracks positions gives the 10th, 25th, 50th, 75th and 90th percentiles by year. The view that tracks countries gives the change in adequacy for the countries occupying the lowest and highest quintiles at the turning point, together with quintile transition matrices for the two sub-periods. The two answer different questions and, as Section 3.4 shows, give different answers. Because the indicator is published rounded to whole numbers, ties occur at the quintile boundaries, and since ties are not split the groups are not exactly equal in size: for 2008 the quintiles contain, from the bottom, 32, 30, 30, 30 and 28 countries, because three countries share the value 107 at the 20th percentile and three share 131 at the 80th. The transition matrices were constructed under the same rule for the first and last year of each sub-period.

Temporal shape was evaluated using three continuous alternatives fitted to the annual dispersion series: a two-segment V, a decline–plateau–increase model whose middle slope is constrained to zero, and an unrestricted three-segment model. Break years were selected by grid search. Models were compared using AICc, BIC and leave-one-year-out prediction error. Because observations are overlapping three-year averages, coefficient uncertainty uses Newey–West covariance with two lags, and model-selection stability is assessed with 2000 moving-block bootstrap samples of length three. The former two-segment specification is retained only as a benchmark.

Country-level panel estimates are retained as supporting distributional descriptions rather than as independent break tests. Country clustering accommodates dependence within countries but cannot remove the mechanical serial dependence created by overlapping three-year averages or the search uncertainty in estimated break dates. The time-series model comparison and three-year block bootstrap therefore provide the primary evidence about temporal shape.

Two points about inference should be stated at the outset. First, the three-year moving average induces serial dependence, which is addressed with two-lag Newey–West covariance and a three-year moving-block bootstrap. Second, break locations are selected from the data. We therefore compare complete model forms and report periods rather than treating a selected year as a fixed hypothesis. These procedures support a decline–near-plateau–widening sequence, not a universal two-segment V.

### 2.4. Factors Associated with the Divergence

The change in adequacy between 2008 and 2023 was related descriptively to cereal import dependence, the food import bill, income, institutional quality and political stability. The complete-covariate model contains 100 countries. A reduced model using only stability change and baseline adequacy contains 107 countries and is reported separately from the complete-covariate model. These samples are kept distinct throughout. Because political stability incorporates violence and conflict and correlates with institutional quality (r = 0.79; variance-inflation factor 2.70), their joint coefficients are not interpreted as separate mechanisms. We emphasise unadjusted co-movement, rank correlations and single-construct sensitivity models rather than a causal governance effect.

Two features of this cross-section were examined directly rather than assumed away. Because the change in political stability and the change in adequacy are measured over overlapping windows, adequacy change was also related to a pre-crisis change in stability, between 2000–2004 and 2005–2009, which is complete before the outcome window opens. Because complete governance data are available for only 100 of the 150 panel countries, the countries lost are reported and the model is re-estimated on the full panel with a reduced set of covariates. False discovery rates across the family of correlates were controlled with the Benjamini–Hochberg procedure [43].

The analysis was carried out in Python; the code and the processed data are available from the author on request.

## 3. Results

### 3.1. Food Availability Improved Almost Everywhere

Across the 150 countries, mean dietary energy supply adequacy rose from 113.8% of requirements in 2001 to 124.8% in 2023, and the median from 113.5 to 125.5. The number of countries whose supply does not cover average requirements fell from 31 in 2001 to 8 in 2013. By 2023 it is back at 12. Country by country, 110 of the 150 ended the period above their 2008 value, 5 were unchanged and 35 were below it.

### 3.2. Countries Stopped Converging and Then Diverged

**Figure 1:**
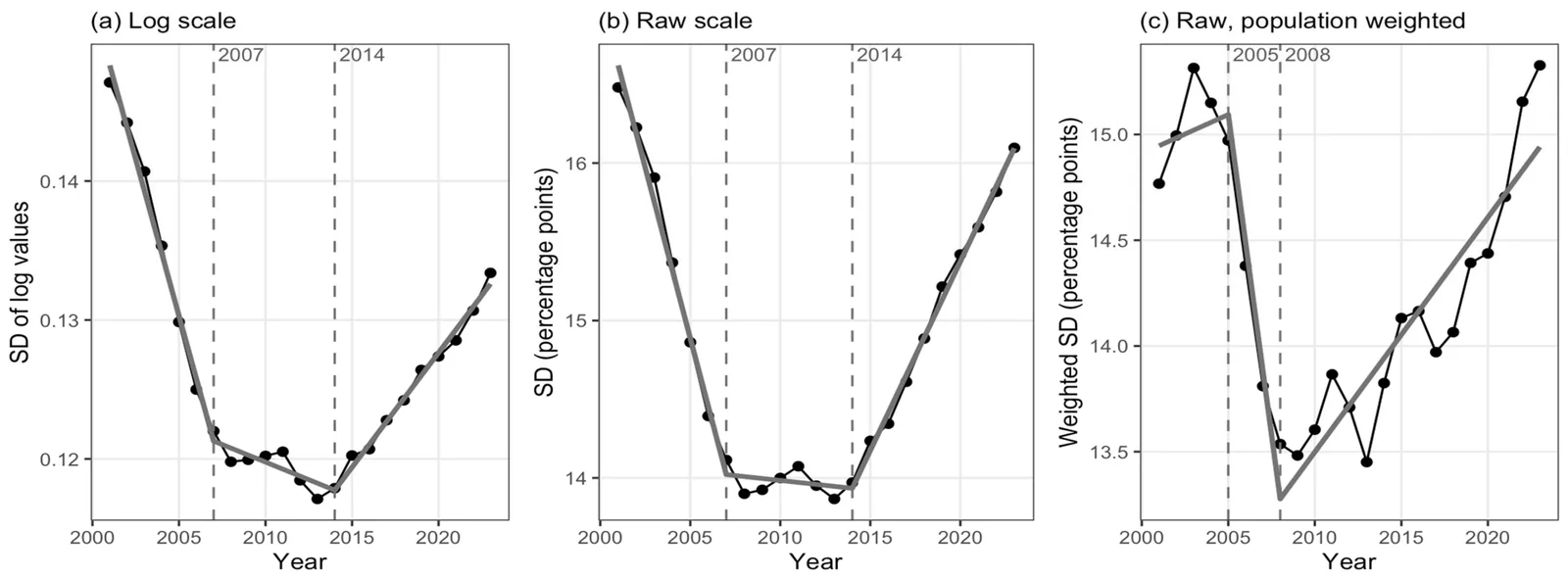
Cross-country distribution of dietary energy supply adequacy, 150 countries, 2001–2023. Points are annual dispersion; grey lines are fitted continuous three-segment models, and dashed lines mark the two estimated breaks in each panel. (**a**) Standard deviation of log values. (**b**) Standard deviation on the raw scale. (**c**) Population-weighted standard deviation on the raw scale.

The dispersion series is better described by three phases than by a two-segment V: rapid narrowing through the late 2000s, a near-plateau extending to the mid-2010s, and widening thereafter. The unrestricted three-segment model selects slope changes in 2007 and 2014, with slopes of −0.00451, −0.00051 and +0.00165. The first break marks deceleration, not the minimum; the shallow negative middle slope allows the observed minimum to occur later, in 2013, immediately before the estimated 2014 shift to positive growth. The model outperforms the plateau model by 12.10 AICc units and the V model by 39.75 units; its leave-one-year-out RMSE is 0.00097, compared with 0.00142 and 0.00293. It is selected in 98.7% of three-year moving-block bootstrap samples. Figure 1 reports three-segment fits separately by scale and weighting, whereas Table 2 and Table 4 retain two-segment estimates only as labelled benchmarks; their dates and slopes therefore need not coincide.

The mean distance between the highest and lowest adequacy fifths was 46 percentage points in 2001, narrowed to 38 by 2013, and reopened to 44 by 2023. Raw-scale dispersion fell from 16.5 to 13.9 percentage points and returned to 16.1, reversing 85% of the compression accumulated between 2001 and 2013. The corresponding reversal is 54% on the logarithmic scale. Population weighting reduces the initial compression but not the later increase (Table 2). Replacing the fixed 2008 population weights with fixed totals from 2001, 2013 or 2023 leaves the minimum in 2013 and yields recovery shares of 1.41, 1.42 and 1.39, respectively, compared with 1.42 under the 2008 weights. Thus, the population-weighted result is not specific to the crisis-year weight vector.

The model comparison remains decisive when serial dependence is respected: the three-segment form is selected in 98.7% of moving-block bootstrap samples, while the plateau model is selected in 1.3% and the two-segment V in none. Repeating the analysis on the latest FAOSTAT vintage selects breaks in 2008 and 2014 and again favours the three-segment form (ΔAICc = 6.97 versus the plateau and 27.36 versus the V). Historical values are not identical across vintages, so the archived snapshot and extraction date are part of the replication package.

Beta-convergence remains negative after 2008, but this does not imply sigma-convergence. The beta coefficient describes whether initially lower countries grew faster on average; sigma dispersion can rise simultaneously when country-specific shocks and rank changes increase residual variation. In this sample the post-2008 beta estimate is small and not distinguishable from zero at 5% (*p* = 0.054), while the cross-country spread rises after the mid-2010s. The two statistics answer different questions and are not expected to move mechanically together (Table 3).

Sensitivity checks do not support describing a single global V. Removing the most volatile 10% and 25% of countries shifts the benchmark two-segment turn to 2013 and 2016 and substantially weakens the early slope. This confirms that rapid early convergence is influenced by high-volatility countries. The more flexible three-segment analysis instead identifies a broad middle period and later widening. We therefore describe the full-sample pattern as decline–near-plateau–widening and avoid treating one break date as a universal global trend (Table 4).

### 3.3. The Turning Pattern Is Also Observed in Other Supply Indicators

The turn is not confined to dietary energy. The same FAOSTAT archive carries three further supply indicators that were not used in constructing the analytical panel.

Average protein supply is measured in grams per person per day; it is a different quantity from the energy measure and carries none of the requirement denominator that adequacy does. On 155 countries it gives the same result: dispersion narrows to 2008, reaches its minimum in 2009, and widens thereafter. It has reversed 70% of its earlier compression. Average supply of protein of animal origin shows the same shape with its turn at 2014. A test run directly on the country-year observations confirms the turn for both indicators (Table 5).

Per capita food-supply variability is non-monotonic: its dispersion rises from its 2001 minimum to a peak around 2008–2011 and then falls substantially. Its fitted post-break slope is not distinguishable from zero (*p* = 0.315), and it does not corroborate a convergence–divergence sequence in the level indicators. Variability is a stability dimension, whereas adequacy and protein supply measure levels; their dispersion paths can therefore differ without being combined into a single two-period model. We report the stability series as a separate outcome and do not use it to date or explain the observed 2013 minimum in adequacy dispersion. The undernourishment and protein indicators are also discussed separately, with their shared FAOSTAT provenance made explicit.

Three level indicators place a change in trajectory between 2008 and 2014, but they are not independent datasets and do not establish a single structural break. Earlier research has shown increasing similarity in the crop composition of national food supplies [13]; our results concern dispersion in the level of energy or protein availability and should not be conflated with compositional homogenisation.

### 3.4. The Lower Tail Is Not Stagnant; It Is Being Replenished

**Figure 2:**
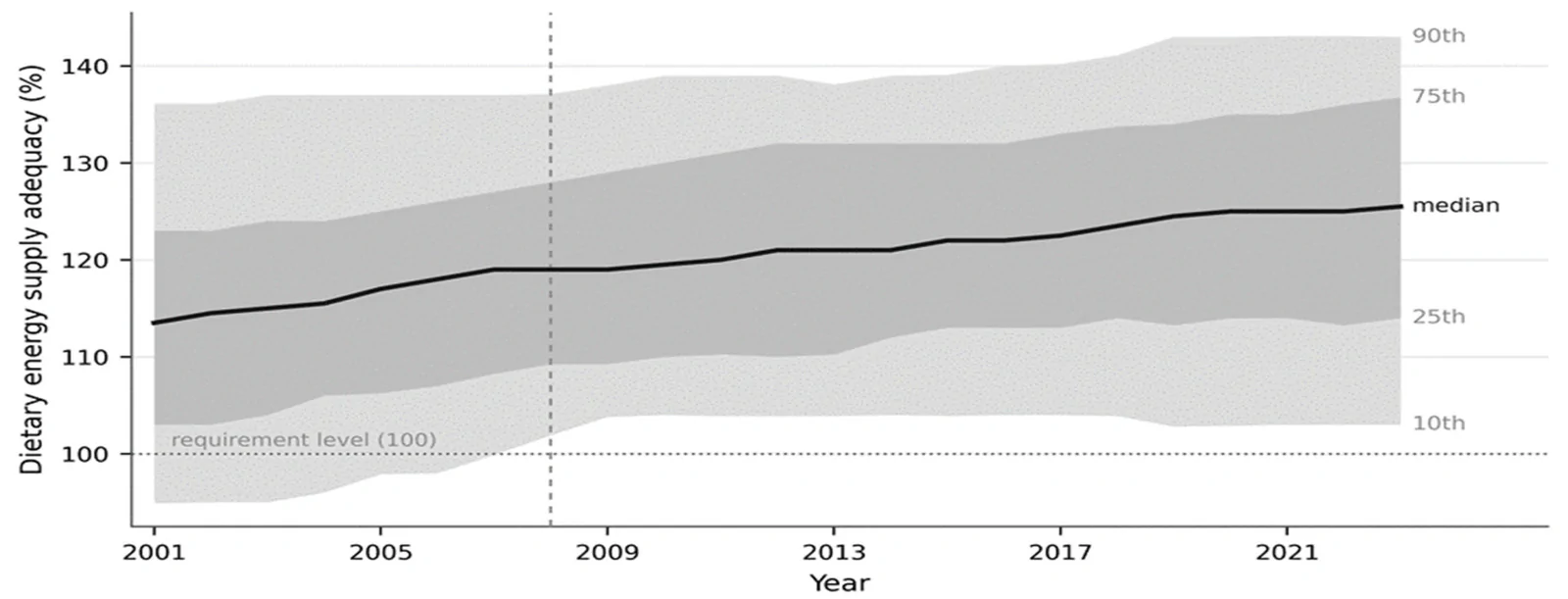
Distribution of dietary energy supply adequacy across 150 countries, 2001–2023. The dark band spans the 25th to 75th percentiles and the light band the 10th to 90th; the line is the median. The dashed vertical line marks the estimated turning point and the dotted horizontal line the requirement level.

Two different comparisons are required. A percentile is an anonymous position in each year’s distribution, whereas the 2008 quintiles follow named countries from a fixed baseline. The 10th percentile rises only 1.1 points between 2008 and 2023, while the countries assigned to the bottom 2008 quintile gain 6.97 points on average. The latter is compared with 4.39 points for the fixed top 2008 quintile, not with a percentile. These quantities therefore cannot be reconciled by rounding alone; they answer different questions. Rounding does, however, limit precision for the 1.1-point percentile movement (as shown in Figure 2).

Among fixed 2008 groups, the 32 countries in the bottom quintile gained 6.97 points on average by 2023, compared with 4.39 points among the 28 countries in the top quintile. Ties at the 2008 boundaries explain the unequal group sizes. These are group means calculated from integer-valued country observations; the difference is 2.58 points and is not created by rounding the reported means. The renewed positional widening is concentrated in the lower half of the distribution. Between 2013 and 2023, the 90th–50th percentile gap increased by 0.4 points, while the 50th–10th percentile gap increased by 5.4 points. Thus, 93.1% of the 5.8-point widening of the 90th–10th percentile gap is attributable to the lower half and 6.9% to the upper half.

Turnover reconciles positional and country-based results, but persistence must be compared over equal durations. Overall quintile persistence is 54.7% in 2001–2008, 58.7% in 2008–2015 and 60.0% in 2016–2023. Bottom-quintile persistence is 73.3%, 70.0% and 73.3%, respectively. The corrected evidence shows continuing turnover, not an increase in mobility.

### 3.5. Divergence Occurred Primarily Within Regions

The conclusion is robust to geographic resolution. Under the seven-region grouping, approximately 85% of the 2008–2023 increase in total log variance is within regions. Repeating the exact decomposition across 22 M49 subregions assigns 78.9% of the increase to the within-subregion component. The between-subregion share is substantial (57.4% in 2008 and 50.3% in 2023) but declines while total dispersion rises. Thus most renewed widening occurs among countries within the same M49 subregion rather than solely between broad world regions.

**Figure 3:**
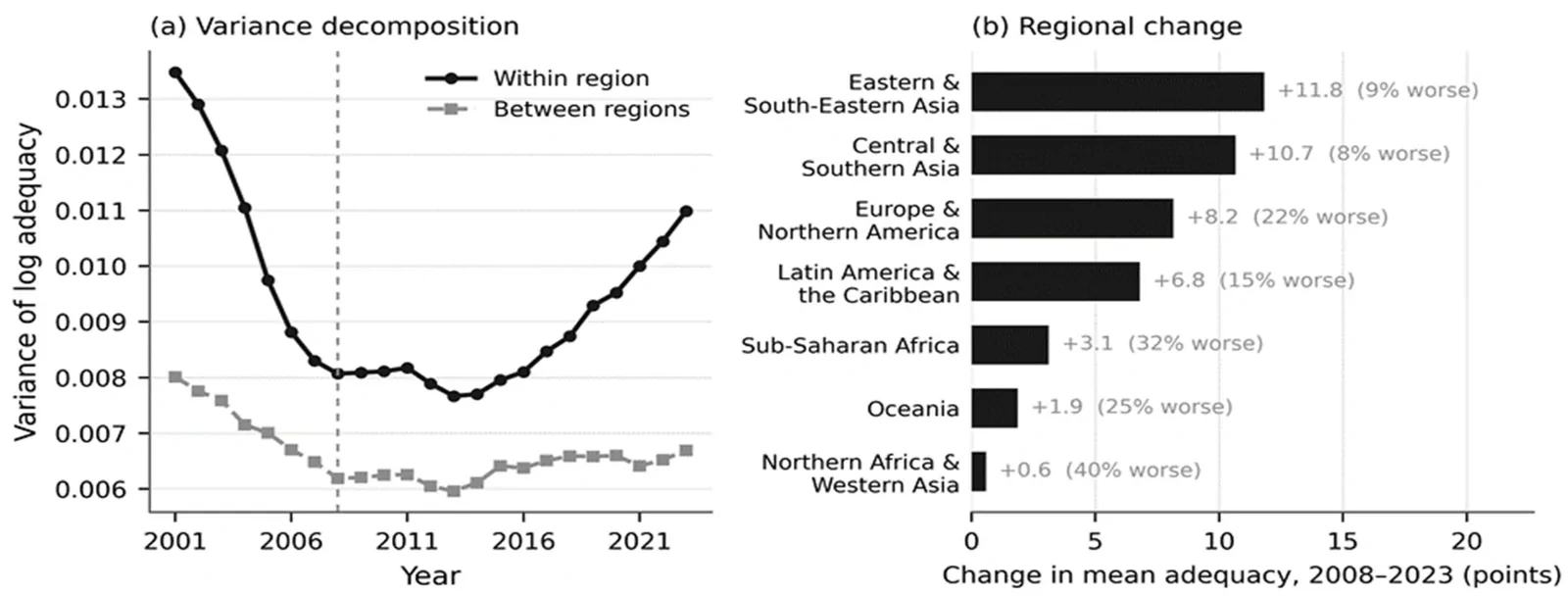
Regional structure of the change. (**a**) Decomposition of the variance of log adequacy into between-region and within-region components. (**b**) Change in mean adequacy by region between 2008 and 2023, with the share of countries in each region whose adequacy fell.

Every region improved on average between 2008 and 2023. The gain was 11.8 points in Eastern and South-Eastern Asia and 10.7 in Central and Southern Asia, against 3.1 in sub-Saharan Africa and 0.6 in Northern Africa and Western Asia. The share of countries within each region whose adequacy fell ranges from 8% in Central and Southern Asia to 32% in sub-Saharan Africa and 40% in Northern Africa and Western Asia. Regional means and regional dispersion moved independently of one another (Figure 3b).

### 3.6. Which Countries Fell Behind?

Thirty-five of the 150 countries closed the period with lower adequacy than in 2008. The seven largest full-sample declines were Syrian Arab Republic (−35 points), Ukraine (−14), Solomon Islands (−13), Jordan, Libya and South Africa (−11 each), and Chad (−10). Nigeria and the Democratic Republic of the Congo declined by −9 and −8 points, respectively, but are not members of the full-sample bottom seven.

In the sample and specifications examined, the trade exposure indicators do not systematically distinguish these countries. Neither the cereal import dependency ratio nor the food import bill as a share of exports is distinguishable from zero, in terms of either the linear coefficient or the rank correlation; the same holds for income per capita. One exception is that the import bill enters the undernourishment column positively and with nominal significance (+1.96; *p* = 0.014); the rank correlation for the same variable, however, is 0.02, and this coefficient does not survive the multiple-testing correction. The divergence between the two measures points to the long right tail of a variable whose 99th percentile is nine times its median rather than to a monotone relationship. No relationship distinguishable from zero was found between baseline adequacy and the change in adequacy (*p* = 0.945); its rank correlation with the change in undernourishment, by contrast, is +0.356, the strongest association in this family. Since the two indicators behave differently in this respect, the role of the baseline level is sensitive to the choice of indicator (Table 6).

**Figure 4:**
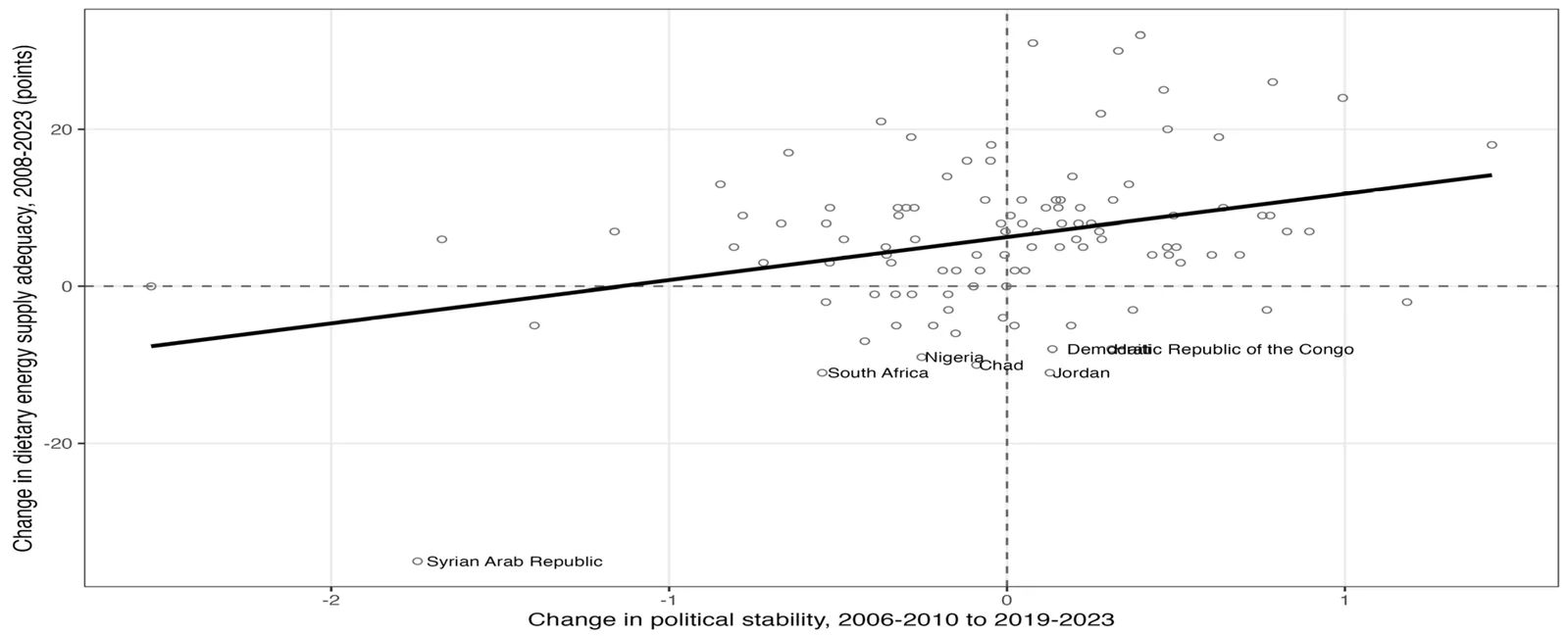
Change in dietary energy supply adequacy between 2008 and 2023 versus contemporaneous change in political stability, *n* = 107. Labels identify the seven largest adequacy declines among countries plotted in this figure, not the seven largest declines in the full 150-country panel. The line and annotated +5.50 slope are unadjusted; Table 6 reports the fully adjusted *n* = 100 model.

In the reduced 107-country sample used for Figure 4, the unadjusted slope relating contemporaneous stability change to adequacy change is +5.50 (*p* = 0.001). The complete-covariate model in Table 6 uses 100 countries and estimates +5.07 (*p* = 0.007). These coefficients are not expected to be identical because the sample and conditioning set differ. They are now labelled separately and are used only as descriptive evidence of contemporaneous co-movement.

Political stability and institutional quality correlate at 0.79 in the 100-country complete-case sample (VIF = 2.70). Because political stability includes violence and conflict, conflict-related food-supply declines can affect both the predictor and outcome during the same window. We therefore do not interpret the two joint coefficients as separate institutional mechanisms and do not privilege their signs. Separate specifications and rank correlations are reported as sensitivity checks, and all language has been changed from explanation to contemporaneous association.

The windows over which the association is measured overlap: the change in stability is computed between 2006–2010 and 2019–2023 and the change in adequacy between 2008 and 2023. When a measure completed before the crisis is used instead—the change in stability between 2000–2004 and 2005–2009—no relationship distinguishable from zero is found in this sample and specification (*p* = 0.225). This result does not establish that the pre-crisis change in stability is unrelated to the subsequent change in adequacy; it shows only that no statistical support was obtained here. Equally, it cannot be read as evidence that the contemporaneous association is causal. What the data show is that the two series moved together within the same window.

The complete-covariate model covers 100 countries; Figure 4 and the reduced specification cover 107. The 50 excluded from Table 6 include Ukraine, Libya, Solomon Islands and the Democratic Republic of the Congo. Of these, Ukraine, Libya and Solomon Islands also lack the stability-change observations required for Figure 4, whereas the Democratic Republic of the Congo enters Figure 4 but not Table 6. Figure 4 labels the seven largest declines within its plotted *n* = 107 sample: Syrian Arab Republic, Jordan, South Africa, Chad, Nigeria, Democratic Republic of the Congo and Haiti. These sample definitions are now stated in the text, table note and caption.

## 4. Discussion

The distribution follows a three-phase pattern rather than a simple V: rapid convergence through the late 2000s, little net change through the mid-2010s, and widening thereafter. This interpretation is preferred by AICc, BIC, leave-one-year-out prediction and a three-year moving-block bootstrap, and it is reproduced in the latest FAOSTAT vintage. The result is therefore not tied to a single 2008 breakpoint, although the magnitude of early convergence remains sensitive to volatile countries.

The lower tail combines weak positional improvement with movement of named countries. Between 2008 and 2023, 18 of the original 30 bottom-quintile countries remained, 12 exited and 12 entered. Entrants were Chad, Comoros, The Gambia, Jordan, Mozambique, Nigeria, Sao Tome and Principe, Solomon Islands, Suriname, Syrian Arab Republic, Uganda and Ukraine. Exits were Angola, Bangladesh, Botswana, Bulgaria, Djibouti, Dominican Republic, Ethiopia, India, Mongolia, Republic of Moldova, Tajikistan and United Republic of Tanzania. Short-run volatility can change membership near a quintile boundary, but it does not account for most recent entry: 10 of the 12 entrants were already in the bottom quintile in every year from 2021 to 2023, while Nigeria and Ukraine entered only in 2023. Among all 30 countries in the 2023 bottom quintile, 27 remained there throughout 2021–2023. Fixed 2008 bottom-quintile countries gained more than fixed top-quintile countries, while equal-duration transition matrices show similar rather than declining persistence. The evidence supports substantial turnover but not a general increase in mobility. Monitoring should therefore report both anonymous positions, multi-year persistence and transitions of named countries.

The timing does not identify a particular crisis. The unrestricted model places changes in 2007 and 2014, while trimming volatile countries moves two-segment estimates as late as 2016. The year 2013 is the observed minimum of the dispersion series, not an estimated structural-break date; it is retained only for descriptive accounting of the preceding compression and subsequent recovery. The 2010 boundary between old and new FAO Food Balance Sheet methods is also relevant: FAO applied the new methodology retrospectively within 2010–2019 but not to the full pre-2010 series [39]. The latest-vintage replication preserves the three-phase form, yet historical revisions average about one adequacy point and the final overlapping window is not identical to the preceding window. We therefore interpret the result as a descriptive period pattern, not as a precisely dated structural break caused by a single event.

Protein-supply indicators show related changes in dispersion, but all derive from the FAOSTAT food-balance system and are not independent replications. Food-supply variability does not show the claimed V: it peaks around 2008–2011 and then declines, with an insignificant fitted post-break slope. The manuscript now distinguishes this stability measure from the level indicators and relates the analysis to prior work on compositional homogenisation [13].

Geographic decomposition supports a predominantly within-area interpretation without relying on only seven broad groups. The within component explains approximately 85% of the 2008–2023 increase under seven regions and 78.9% under 22 M49 subregions. Regional structure remains important, but the renewed widening is driven mainly by differences among countries inside the same subregion.

That the trade exposure indicators show no distinguishing association bears on a long-standing policy debate. In the sample and specifications examined, neither the cereal import dependency ratio nor the burden of the food import bill systematically distinguishes the countries that fell behind, in either outcome indicator or by either method. The proposition that dependence on imported food is the principal source of national food-system fragility has motivated self-sufficiency strategies in many countries, and the counter-argument that protectionism may reduce food security rather than raise it has been made from national evidence [38,44]. Import dependence is of course a real channel: the transmission of international prices to domestic markets is well established, and its magnitude depends on trade structure and self-sufficiency [15,16,17,18,19,20,21]. Work published in this journal has traced supply risk to the architecture of cereal trade networks and to technological conditions in price and supply dynamics [45,46]. The present finding does not question the existence of that channel; it shows only that, over the period and for the indicators examined, the size of trade exposure was not systematically related to cross-country change in adequacy. This does not mean that price and supply shocks are not transmitted through trade.

Contemporaneous political-stability change has the most consistent descriptive association with adequacy change, but it is not interpreted as a causal or institutional effect. The WGI measure explicitly incorporates violence and conflict; conflict can simultaneously reduce food supply and the stability score. Its high correlation with institutional quality further prevents separate structural interpretation. The result describes co-movement within the same period and differs between the *n* = 107 unadjusted figure and *n* = 100 adjusted model.

Because the association is contemporaneous, no direction can be inferred. A stability measure completed before the outcome window is not statistically associated with subsequent adequacy change (*p* = 0.225). Conflict, reverse causation and common shocks can operate simultaneously. We therefore remove language presenting political stability as an explanation and report it only as a characteristic that co-moved with adequacy in the observed sample.

Read together, the two findings raise the question of which indicators are used to assess food-system vulnerability. Exposure measures such as import dependence describe the channel through which an external disturbance reaches a country. In this sample and over this period, what was associated with cross-country change in adequacy was not the width of that channel but contemporaneous change in an indicator of state functioning. That observation sits more easily with the capacity dimension of resilience frameworks [27,28,29] and with the agency dimension of broader food security accounts [47] than with trade exposure. The finding does not displace the instruments recommended by the price-policy literature: transparent stock management, predictable border measures and scalable social protection remain well founded [48,49,50,51,52,53,54,55,56]. How much information trade-share indicators carry for ranking vulnerability is a separate question that would require an out-of-sample predictive comparison; no such comparison was made in this study.

There is also a monitoring implication. Food security reporting is organised around levels and regional aggregates, neither of which makes the pattern documented here visible. A dispersion statistic computed on an indicator that is already published annually for almost every country would make the distributional trajectory traceable at no additional data cost.

Three groups of limitations qualify the findings. The first concerns measurement. Dietary energy supply adequacy is an availability measure computed from food balance sheets; it is silent on distribution within countries, on diet quality and on economic access, and availability and access need not move together [34,35,57]. The results here therefore concern national supply rather than household-level food security. The denominator of the indicator is the average dietary energy requirement, which depends on the age, sex and body-size composition of the population, so a country’s adequacy can rise as its population ages without any change in supply. That the protein supply indicators, which carry no requirement denominator, show the same turn is evidence against a purely demographic reading, but a full separation would require the supply and requirement series individually. Because the indicator is published rounded to whole numbers, statistics based on a single percentile are sensitive; this is why the lower tail is characterised in Section 3.4 by country groups rather than by a single percentile.

The second group concerns timing, data construction and inference. The observations are overlapping three-year averages, the final 2023 point represents 2022–2024, and historical FAOSTAT values are revised across releases. The primary temporal analysis therefore compares two-segment, plateau and three-segment forms, uses two-lag Newey–West covariance, and resamples residuals in three-year blocks. A latest-vintage re-extraction recovers the three-phase pattern but does not reproduce every historical value. The archived data snapshot is essential for exact replication.

The third group concerns samples and associations. The main panel contains 150 countries, the complete governance model 100, and the reduced stability model and Figure 4 use 107. These denominators are now reported separately. Political stability and institutional quality correlate at 0.79, and stability includes violence, so their contemporaneous coefficients cannot identify separate mechanisms. The design remains descriptive and none of these associations is interpreted causally.

Four extensions follow. Why the turn in animal protein supply arrives six years later may have to do with shifts in the composition of supply and merits separate examination. Decomposing adequacy into its supply and requirement components would establish how much of the divergence is demographic. Event-based conflict data, dated independently of the outcome, would allow the stability association to be tested for precedence rather than co-movement. And identifying what separates the countries that climbed out of the lowest quintile from those that fell into it speaks directly to the turnover mechanism documented here; this is the sharpest question the distributional result raises.

## 5. Conclusions

Across 150 countries observed from 2001 to 2023, cross-country dispersion in dietary energy supply adequacy is best described by rapid narrowing, a near-plateau, and renewed widening. The unrestricted three-segment model selects 2007 and 2014 and decisively outperforms a two-segment V and constrained plateau. The latest FAOSTAT vintage reproduces the three-phase form, although historical revisions and overlapping terminal windows mean that exact values are release-specific. The finding is therefore a sample-based distributional pattern, not a universal V or a break attributable to one year.

The renewed widening is predominantly within geographic groups: 85% of the 2008–2023 increase under seven regions and 78.9% under 22 M49 subregions. Fixed bottom-quintile countries improved faster than fixed top-quintile countries, but equal-duration transition matrices do not show increasing mobility. Position-based and country-based results should therefore be reported together.

Political stability and adequacy co-move contemporaneously, but the association is not interpretable as a governance effect because the stability measure contains conflict, overlaps the outcome window, and is correlated with institutional quality. Trade exposure and income do not show consistent associations in the examined specifications.

These findings suggest several specific implications for food-security monitoring and policy. First, international and national monitoring frameworks should complement global and regional averages with a transparent cross-country dispersion measure and routinely report changes in the lower tail of the distribution. Second, because most of the renewed widening occurs within rather than between regions, policy responses should not rely solely on broad regional classifications. Monitoring systems should identify countries that persist in, enter, or exit the lowest adequacy group and use these country-level trajectories to guide more targeted assessment and support. Third, the concentration of recent widening below the median indicates that particular attention should be directed toward countries experiencing persistent or repeated deterioration in dietary energy supply adequacy, rather than assuming that improvements in regional means imply broadly shared progress. Finally, because the observed association between political stability and adequacy is descriptive rather than causal, it should be used as a signal for closer vulnerability assessment rather than as a basis for prescribing governance interventions. For reproducible monitoring, data vintages should be preserved and alternative temporal forms should be compared rather than imposing a single turning year or a two-segment V.

## Figures and Tables

**Table 1:** Indicators, balanced samples and coverage.

Indicator	Balanced Countries	Balanced Years	Observations	Mean (First Year)	Mean (Last Year)	Min	Max
Dietary energy supply adequacy (%)	150	2001–2023	3450	113.8	124.8	71.0	167.0
Prevalence of undernourishment (%)	159	2001–2023	3657	14.5	9.5	<2.5 (published bound)	72.5
Moderate or severe food insecurity (%)	92	2015–2023	828	24.5	27.9	1.1	89.8

Notes: Balanced panels require a complete annual series over the stated period. Means are cross-country means in the first and last year of that period; minimum and maximum are over all available observations of the indicator.

**Table 2:** Four measures of the adequacy distribution, with the estimated turning point and the share of the earlier compression reversed.

Dispersion Measure	Two-Segment Benchmark Turn	Slope Before	Slope After	Compression to Minimum	Minimum Year	Share of Compression Reversed
Unweighted, log scale	2008	−0.0042 (<0.001)	+0.0009 (<0.001)	−20.4%	2013	0.54
Unweighted, raw scale	2009	−0.3628 (<0.001)	+0.1637 (<0.001)	−15.9%	2013	0.85
Population weighted, log scale	2007	−0.0019 (0.053)	+0.0004 (<0.001)	−11.7%	2013	0.73
Population weighted, raw scale	2009	−0.2216 (0.004)	+0.1160 (<0.001)	−8.9%	2013	1.42

Notes: Dispersion is the cross-country standard deviation of adequacy in each year, on the stated scale. Population weights are 2008 totals and are held fixed. The reported turn is retained as a two-segment benchmark only; the primary analysis compares the complete two-segment V, plateau and unrestricted three-segment forms using AICc, BIC, leave-one-year-out prediction and a three-year moving-block bootstrap. The share of compression reversed is the rise from the observed minimum divided by the fall from the first year to the minimum; a value above one means dispersion in 2023 exceeds its 2001 level. The logarithmic scale records a larger compression and a smaller reversal than the raw scale because the cross-country mean rises by 9.6% over the period and the logarithm places a rising mean in the denominator.

**Table 3:** Cross-country speed of catching up, by indicator and period.

Indicator	Period	Beta	SE	*p*	R^2^	Countries
Dietary energy supply adequacy	2001–2023	−1.692	0.245	<0.001	0.244	150
Dietary energy supply adequacy	2001–2008	−4.057	0.456	<0.001	0.349	150
Dietary energy supply adequacy	2008–2023	−0.705	0.364	0.054	0.025	150
Prevalence of undernourishment	2001–2023	−1.208	0.241	<0.001	0.138	159
Prevalence of undernourishment	2001–2009	−2.508	0.489	<0.001	0.143	159
Prevalence of undernourishment	2009–2023	−0.815	0.328	0.014	0.038	159
Moderate or severe food insecurity	2015–2023	−0.368	0.62	0.554	0.004	92
Moderate or severe food insecurity	2015–2018	1.23	0.958	0.203	0.018	92
Moderate or severe food insecurity	2018–2023	−1.004	0.699	0.154	0.022	92

Notes: Beta is the coefficient from a cross-country regression of the average annual log growth rate of the indicator on its initial log level. A negative value indicates that initially lower countries improved faster. Periods are split at the turning point estimated for each indicator. The experience-scale series begins in 2015 and is reported for completeness only.

**Table 4:** Robustness of the estimated turning point.

Specification	Countries	Two-Segment Benchmark Turn	Slope Before	*p* (Before)	Slope After	*p* (After)	Panel Slope Change	*p* (Panel)	Panel Change at 2008	*p* (at 2008)
Preferred: balanced panel, log dispersion	150	2008	−0.00422	<0.001	+0.00089	<0.001	+0.00469	<0.001	—	—
Conflict-affected countries excluded	139	2008	−0.00458	<0.001	+0.00072	<0.001	+0.00428	<0.001	—	—
Ten highest-adequacy countries excluded	140	2008	−0.00370	<0.001	+0.00110	<0.001	+0.00455	<0.001	—	—
Ten lowest-adequacy countries excluded	140	2008	−0.00530	<0.001	+0.00121	<0.001	+0.00495	<0.001	—	—
Low- and middle-income regions only	90	2008	−0.00409	<0.001	+0.00117	<0.001	+0.00517	<0.001	—	—
Dispersion measured as iqr over median	150	2014	+0.00034	0.576	+0.00248	0.003	—	—	—	—
Dispersion measured as p90 minus p10	150	2013	−0.72088	<0.001	+0.69364	<0.001	—	—	—	—
Dispersion measured as cv	150	2009	−0.00394	<0.001	+0.00096	<0.001	—	—	—	—
Unbalanced panel, at least 18 years	152	2008	−0.00399	<0.001	+0.00070	<0.001	+0.00436	<0.001	—	—
Most volatile 10% excluded	135	2013	−0.00215	<0.001	+0.00147	<0.001	+0.00317	<0.001	+0.00387	<0.001
Most volatile 25% excluded	114	2016	−0.00111	<0.001	+0.00160	<0.001	+0.00211	0.011	+0.00214	0.011

Notes: Each row re-estimates the turning point and the two segment slopes under the stated change. The panel columns are the country-clustered slope change described in the statistical methods, freely estimated and then holding the turning point at 2008; they are not computed for the alternative dispersion measures. Volatility is the mean absolute annual change in adequacy. Cells that do not apply are shown as an em dash. The conflict-affected states excluded are Syrian Arab Republic, Ukraine, Libya, Afghanistan, Somalia, Haiti, Democratic Republic of the Congo, Chad, Mali, Venezuela (Bolivarian Republic of) and Iraq.

**Table 5:** Dispersion turning points in four FAO supply indicators.

Indicator	Countries	Period	Two-Segment Benchmark Turn	Minimum	Slope Before	Slope After	Panel Slope Change	Share Reversed
Dietary energy supply adequacy	150	2001–2023	2008	2013	−0.0042 (<0.001)	+0.0009 (<0.001)	<0.001	0.54
Average protein supply	155	2001–2022	2008	2009	−0.0028 (<0.001)	+0.0011 (<0.001)	0.027	0.70
Average supply of animal protein	155	2001–2022	2014	2015	−0.0062 (<0.001)	+0.0026 (<0.001)	0.004	0.29
Per capita food supply variability	140	2001–2023	2012	2001	+0.0255 (0.004)	+0.0034 (0.315)	0.012	—

Notes: Each row applies the same segmented fit to the annual standard deviation of log values on a balanced panel of the stated indicator. Panel slope change is the country-clustered test described in the note to the statistical methods. Food supply variability reaches its minimum in the first year of the panel, so no share of an earlier compression can be computed. The protein series are published through 2022.

**Table 6:** Factors associated with the change in food security indicators between the turning point and 2023.

Variable	Coefficient (Adequacy)	*p* (Adequacy)	Spearman Rho (Adequacy)	Spearman *p* (Adequacy)	Coefficient (Undernourishment)	*p* (Undernourishment)	Spearman Rho (Undernourishment)	Spearman *p* (Undernourishment)
Political stability, level	+4.016	0.024	−0.018	0.853	−2.490	0.033	+0.197	0.037
Political stability, change	+5.068	0.007	+0.255	0.008	−3.371	0.003	−0.285	0.002
Institutional quality	−4.836	0.013	−0.033	0.736	+1.320	0.158	+0.224	0.017
Food import bill (log, share of exports)	−1.035	0.433	−0.064	0.433	+1.964	0.014	+0.017	0.835
Cereal import dependence	−0.009	0.587	−0.118	0.166	−0.009	0.160	+0.037	0.659
Log GDP per capita	+0.694	0.692	+0.100	0.229	+1.333	0.258	+0.138	0.086
Baseline adequacy	−0.007	0.945	−0.077	0.350	+0.060	0.329	+0.356	<0.001

Notes: Dependent variables are the country-level change in the indicator between the turning point and 2023. Correlates are averaged over the five years centred on the turning point; the stability change compares 2019–2023 with 2006–2010. Standard errors are heteroskedasticity-robust. *n* = 100 countries with complete governance data. Institutional quality and political stability correlate at 0.79 in this sample, so their coefficients are not separately identified; the reduced specifications reports the reduced specifications.

## Data Availability

The processed analytical dataset, the construction and estimation code and all output tables are available from the corresponding author on reasonable request. The source indicators are publicly accessible from the FAOSTAT Data portal (https://www.fao.org/faostat/) (accessed on 28 August 2026), the World Bank Worldwide Governance Indicators portal (https://www.worldbank.org/en/publication/worldwide-governance-indicators) (accessed on 28 August 2026) and the World Development Indicators portal (https://databank.worldbank.org/source/world-development-indicators) (accessed on 28 August 2026).

## Abbreviations

The following abbreviations are used in this manuscript:

FAO	Food and Agriculture Organization of the United Nations
FAOSTAT	Food and Agriculture Organization Corporate Statistical Database
GDP	Gross Domestic Product
SDG	Sustainable Development Goal

## References

[1] Food and Agriculture Organization of the United Nations, International Fund for Agricultural Development, United Nations Children’s Fund, World Food Programme, World Health Organization (2025). The State of Food Security and Nutrition in the World 2025.

[2] Naylor R.L., Falcon W.P. (2010). Food security in an era of economic volatility. Popul. Dev. Rev..

[3] Headey D. (2011). Rethinking the global food crisis: The role of trade shocks. Food Policy.

[4] Headey D., Fan S. (2010). Reflections on the Global Food Crisis: How Did It Happen? How Has It Hurt? And How Can We Prevent the Next One?.

[5] Arndt C., Diao X., Dorosh P., Pauw K., Thurlow J. (2023). The Ukraine war and rising commodity prices: Implications for developing countries. Glob. Food Secur..

[6] Ivanic M., Martin W. (2008). Implications of higher global food prices for poverty in low-income countries. Agric. Econ..

[7] Anríquez G., Daidone S., Mane E. (2013). Rising food prices and undernourishment: A cross-country inquiry. Food Policy.

[8] Amolegbe K.B., Upton J., Bageant E., Blom S. (2021). Food price volatility and household food security: Evidence from Nigeria. Food Policy.

[9] Matz J.A., Kalkuhl M., Abegaz G.A. (2015). The short-term impact of price shocks on food security—Evidence from urban and rural Ethiopia. Food Secur..

[10] Akter S., Basher S.A. (2014). The impacts of food price and income shocks on household food security and economic well-being: Evidence from rural Bangladesh. Glob. Environ. Chang..

[11] Nag A., Přívara A., Gavurová B., Pradhan J. (2023). Does club convergence matter in health outcomes? Evidence from Indian states. BMC Public Health.

[12] Maynou L., Sáez M., Bacaria J., Lopez-Casasnovas G. (2014). Health inequalities in the European Union: An empirical analysis of the dynamics of regional differences. Eur. J. Health Econ..

[13] Khoury C.K., Bjorkman A.D., Dempewolf H., Ramirez-Villegas J., Guarino L., Jarvis A., Rieseberg L.H., Struik P.C. (2014). Increasing homogeneity in global food supplies and the implications for food security. Proc. Natl. Acad. Sci. USA.

[14] Bell W., Lividini K., Masters W.A. (2021). Global dietary convergence from 1970 to 2010 altered inequality in agriculture, nutrition and health. Nat. Food.

[15] Ceballos F., Hernandez M.A., Minot N., Robles M. (2017). Grain price and volatility transmission from international to domestic markets in developing countries. World Dev..

[16] Emediegwu L.E., Rogna M. (2024). Agricultural commodities’ price transmission from international to local markets in developing countries. Food Policy.

[17] Bekkers E., Brockmeier M., Francois J., Yang F. (2017). Local food prices and international price transmission. World Dev..

[18] Luo P., Tanaka T. (2021). Food import dependency and national food security: A price transmission analysis for the wheat sector. Foods.

[19] Guo J., Tanaka T. (2019). Determinants of international price volatility transmissions: The role of self-sufficiency rates in wheat-importing countries. Palgrave Commun..

[20] Dissanayake J. (2016). Agricultural price transmission elasticity: Importance of controlling for unobserved common factors. Agric. Econ..

[21] Baltzer K., Pinstrup-Andersen P. (2015). International to domestic price transmission in fourteen developing countries during the 2007–2008 food crisis. Food Price Policy in an Era of Market Instability: A Political Economy Analysis.

[22] Fotakis E.A., Kontele I., Tzoutzou M., Grammatikopoulou M.G., Arvanitaki E., Sergentanis T.N., Kotrokois K., Kornarou E., Vassilakou T. (2024). Food insecurity in Greece and across the globe: A narrative literature review. Foods.

[23] George J., Adelaja A.O., Weatherspoon D.D. (2020). Armed conflicts and food insecurity: Evidence from Boko Haram’s attacks. Am. J. Agric. Econ..

[24] Weldegiargis A.W., Abebe H.T., Abraha H.E., Abrha M.M., Tesfay T.B., Belay R.E., Araya A.A., Gebregziabher M.B., Godefay H., Mulugeta A. (2023). Armed conflict and household food insecurity: Evidence from war-torn Tigray, Ethiopia. Confl. Health.

[25] Bellemare M.F. (2015). Rising food prices, food price volatility, and social unrest. Am. J. Agric. Econ..

[26] Berazneva J., Lee D.R. (2013). Explaining the African food riots of 2007–2008: An empirical analysis. Food Policy.

[27] Tendall D.M., Joerin J., Kopainsky B., Edwards P., Shreck A., Le Q.B., Kruetli P., Grant M., Six J. (2015). Food system resilience: Defining the concept. Glob. Food Secur..

[28] Béné C. (2020). Resilience of local food systems and links to food security—A review of some important concepts in the context of COVID-19 and other shocks. Food Secur..

[29] Constas M.A., Béné C., Devereux S. (2023). Food security and resilience: The potential for coherence and the reality of fragmented applications in policy and research. Resilience and Food Security in a Food Systems Context.

[30] Liu Y., Chen J., Cheng W., Wang X., Pan T., Liu J., Lu Y., Zhang E., Huang S., Zhang J. (2026). Multidimensional synergistic adaptation enhances the systemic resilience in China’s food security. Natl. Sci. Rev..

[31] Yang G., Li H., Zhao X., Yang J., Guo H., Wei D., Huo L. (2026). Optimized Allocation of Irrigation Water Resources Based on Uncertainty: Model Construction and Dynamic Regulation Mechanism. Water.

[32] Yang G.Q., Li M., Guo P. (2022). Monte Carlo-Based Agricultural Water Management under Uncertainty: A Case Study of Shijin Irrigation District, China. J. Environ. Inform..

[33] Liu Y., Zhang E., Pan T., Chen J., Gao Y., Peñuelas J. (2025). Anthropogenic adaptation measures expand suitable area for highland barley in Tibetan Plateau. Sci. Bull..

[34] Manikas I., Ali B.M., Sundarakani B. (2023). A systematic literature review of indicators measuring food security. Agric. Food Secur..

[35] Hossain M.B., Long M.A., Stretesky P.B. (2021). Welfare state spending, income inequality and food insecurity in affluent nations: A cross-national examination of OECD countries. Sustainability.

[36] Gnedeka K.T., Wonyra K.O. (2023). New evidence in the relationship between trade openness and food security in Sub-Saharan Africa. Agric. Food Secur..

[37] Fusco G., Coluccia B., De Leo F. (2020). Effect of trade openness on food security in the EU: A dynamic panel analysis. Int. J. Environ. Res. Public Health.

[38] Macdiarmid J.I., Clark H., Whybrow S., de Ruiter H., McNeill G. (2018). Assessing national nutrition security: The UK reliance on imports to meet population energy and nutrient recommendations. PLoS ONE.

[39] Food and Agriculture Organization of the United Nations (2022). Key Differences Between New and Old Food Balance Sheet Methodology.

[40] Kaufmann D., Kraay A. (2024). The Worldwide Governance Indicators: Methodology and 2024 Update.

[41] Barro R.J., Sala-i-Martin X. (1992). Convergence. J. Polit. Econ..

[42] Quah D. (1993). Empirical cross-section dynamics in economic growth. Eur. Econ. Rev..

[43] Benjamini Y., Hochberg Y. (1995). Controlling the false discovery rate: A practical and powerful approach to multiple testing. J. R. Stat. Soc. Ser. B.

[44] Erokhin V. (2017). Self-sufficiency versus security: How trade protectionism challenges the sustainability of the food supply in Russia. Sustainability.

[45] Zhang C., Yang Y., Feng Z., Xiao C., Lang T., Du W., Liu Y. (2021). Risk of global external cereals supply under the background of the COVID-19 pandemic: Based on the perspective of trade network. Foods.

[46] Doran N.M. (2025). Boosting food system stability through technological progress in price and supply dynamics. Foods.

[47] Clapp J., Moseley W.G., Burlingame B., Termine P. (2022). Viewpoint: The case for a six-dimensional food security framework. Food Policy.

[48] Serra T., Gil J.M. (2013). Price volatility in food markets: Can stock building mitigate price fluctuations?. Eur. Rev. Agric. Econ..

[49] Anderson K. (2014). The intersection of trade policy, price volatility, and food security. Annu. Rev. Resour. Econ..

[50] Martin W., Anderson K. (2012). Export restrictions and price insulation during commodity price booms. Am. J. Agric. Econ..

[51] Anderson K. (2012). Government trade restrictions and international price volatility. Glob. Food Secur..

[52] Giordani P.E., Rocha N., Ruta M. (2016). Food prices and the multiplier effect of trade policy. J. Int. Econ..

[53] Dietrich S., Schmerzeck G. (2019). Cash transfers and nutrition: The role of market isolation after weather shocks. Food Policy.

[54] Ahmed A., Hoddinott J., Roy S. (2023). Private transfers, public transfers, and food insecurity during the time of COVID-19: Evidence from Bangladesh. Appl. Econ. Perspect. Policy.

[55] Kalkuhl M., von Braun J., Torero M., Kalkuhl M., von Braun J., Torero M. (2016). Volatile and extreme food prices, food security, and policy: An overview. Food Price Volatility and Its Implications for Food Security and Policy.

[56] Serra T. (2013). Time-series econometric analyses of biofuel-related price volatility. Agric. Econ..

[57] Castellani D., Tavoni A., D’Odorico P. (2026). An empirical socio-economic indicator of country-level food security. Environ. Res. Lett..

